# Spillover: Animal Infection and the Next Human Pandemic

**DOI:** 10.3201/eid1902.121694

**Published:** 2013-02

**Authors:** Corrie Brown

**Affiliations:** Author affiliation: University of Georgia, Athens, Georgia, USA

Spillover is a single event during which a pathogen from 1 species moves into another species; such movement can result in an outbreak. In 9 chapters, David Quammen chronicles various spillover events by using personal anecdotes and multiple stories to recount these events for the expert and novice alike. He frames the events within an ecologic sense of the pathogen, the host, and the increasing human population. He focuses recurrently on the NBO (next big one) and how, if HIV or Ebola virus were more easily transmissible, no one would remain to read his book.

Quammen’s analogies are superb. Instead of trying to turn the reader into a scientist with dry explanations, he uses analogies that have universal relevance. For viral morphology, Ebola and Hendra virions together would resemble a “capellini in a light sauce of capers.” Mathematical modeling can be appreciated in translation, just as Dostoevsky can be appreciated in translation instead of in the original Russian. Quammen compares combining specific antibodies with their virus to splashing holy water on a witch. Regarding airborne transmission, he says that pathogens can “waft into a nearby village as easily as the pleasant, autumnal smell of smoke from a pile of leaves.” Throughout the book, the subjects of human and animal diseases are “. . . strands of one braided cord.”

The last chapter, “It Depends,” is particularly sobering. If, in an ecologic sense, an outbreak is a rapid and explosive increase in the abundance of a particular species, then maybe humans are the current outbreak in the world. We have become a dense forest; tinder is dry; and the NBO is around the corner.

Who should read this book? Anyone interested in science can enjoy it—those who make their living at the bench, teach, or study—and anyone just looking for a good read.

